# Acibenzolar-*S*-Methyl Reprograms Apple Transcriptome Toward Resistance to Rosy Apple Aphid

**DOI:** 10.3389/fpls.2018.01795

**Published:** 2018-12-12

**Authors:** Romain Warneys, Matthieu Gaucher, Philippe Robert, Sophie Aligon, Sylvia Anton, Sébastien Aubourg, Nicolas Barthes, Ferréol Braud, Raphaël Cournol, Christophe Gadenne, Christelle Heintz, Marie-Noëlle Brisset, Alexandre Degrave

**Affiliations:** ^1^IRHS, INRA, Agrocampus-Ouest, Université d’Angers, SFR 4207 QuaSaV, Beaucouzé, France; ^2^IGEPP, INRA, Agrocampus-Ouest, Université de Rennes 1, Angers, France; ^3^Centre d’Ecologie Fonctionnelle et Evolutive, UMR 5175, CNRS – Université de Montpellier – Université Paul Valery Montpellier 3 – EPHE – IRD, Montpellier, France

**Keywords:** amaranthin-like lectin, acibenzolar-*S*-methyl, *Dysaphis plantaginea*, farnesene, *Malus* × *domestica* (apple), plant resistance inducer, sesquiterpene

## Abstract

Acibenzolar-*S*-methyl (ASM) is a chemical compound, which is able to induce resistance in several model and non-model plants, but the end-players of this induced defense remain ill-defined. Here, we test the hypothesis that treatment with ASM can protect apple (*Malus* × *domestica*) against the rosy apple aphid (*Dysaphis plantaginea*) and investigate the defense molecules potentially involved in resistance. We measured aphid life traits and performed behavioral assays to study the effect of ASM on plant resistance against the aphid, and then combined transcriptomic, bioinformatics, metabolic and biochemical analyses to identify the plant compounds involved in resistance. Plants treated with ASM negatively affected several life traits of the aphid and modified its feeding and host seeking behaviors. ASM treatment elicited up-regulation of terpene synthase genes in apple and led to the emission of (*E,E*)-α-farnesene, a sesquiterpene that was repellent to the aphid. Several genes encoding amaranthin-like lectins were also strongly up-regulated upon treatment and the corresponding proteins accumulated in leaves, petioles and stems. Our results link the production of specific apple proteins and metabolites to the antibiosis and antixenosis effects observed against *Dysaphis plantaginea*, providing insight into the mechanisms underlying ASM-induced herbivore resistance.

## Introduction

Acibenzolar-*S*-methyl (ASM, also called benzo-1,2,3-thiadiazole-7-carbothioic acid *S*-methyl ester or BTH) is a well-known functional analog of salicylic acid (SA), inducing resistance in a number of plant species against a wide range of plant pathogens, at least under controlled conditions (Thakur and Sohal, 2013; Walters et al., 2013). Many studies have demonstrated the capacity of ASM to induce plant defenses related to the systemic acquired resistance (SAR) (Durrant and Dong, 2004; Bektas and Eulgem, 2015), and this compound is frequently used to chemically induce SAR in plant model systems to decipher the SAR molecular network (see for example Wang et al., 2006a).

Several studies have revealed interesting effects of ASM on apple (*Malus* × *domestica*). It efficiently protects plants in greenhouse conditions from the two major apple pathogens *Venturia inaequalis*, the ascomycete fungus responsible for apple scab (Bengtsson et al., 2006; Marolleau et al., 2017) and *Erwinia amylovora*, the enterobacterium responsible for fire blight (Brisset et al., 2000; Baysal and Zeller, 2004; Dugé De Bernonville et al., 2014). Attempts to integrate ASM into protection practices in orchards have also given promising results, either after classical spray application to control apple scab (Marolleau et al., 2017) and fire blight (Maxson-Stein et al., 2002), or after trunk injection to control fire blight (Aćimović et al., 2015). Several molecular studies revealed that ASM application promotes the overexpression of specific defense genes, mainly encoding pathogenesis-related (PR) proteins in apple (Brisset et al., 2000; Ziadi et al., 2001; Maxson-Stein et al., 2002; Dugé De Bernonville et al., 2014; Aćimović et al., 2015; Marolleau et al., 2017).

The use of ASM to control pest insects such as aphids is far less documented. Decrease of insect growth and development on ASM-treated plants has been, however, reported in a few systems including *Myzus persicae*/Arabidopsis (Moran and Thompson, 2001), *Macrosiphum euphorbiae*/tomato (Cooper et al., 2004), and *M. persicae*/tomato (Boughton et al., 2006; Civolani et al., 2010). Chemically induced resistance against aphids merits further investigations, especially as plant resistance to aphids involves salicylate and jasmonate signals as well as subsequent signaling crosstalks (Smith and Chuang, 2014). This is particularly true for ASM against the apple rosy aphid (*Dysaphis plantaginea*) due to the known effect of this particular compound against apple scab and fire blight. These three apple health issues are impacting apple crop at the same period (Spring) and the use of compounds providing a broad spectrum of resistance is an attractive strategy in frame with the current pesticide-reduction policies. Besides, the available apple genome makes the apple/rosy apple aphid interaction an ideal system to investigate the mechanisms involved in plant protection by ASM against insect pests.

**FIGURE 1:**
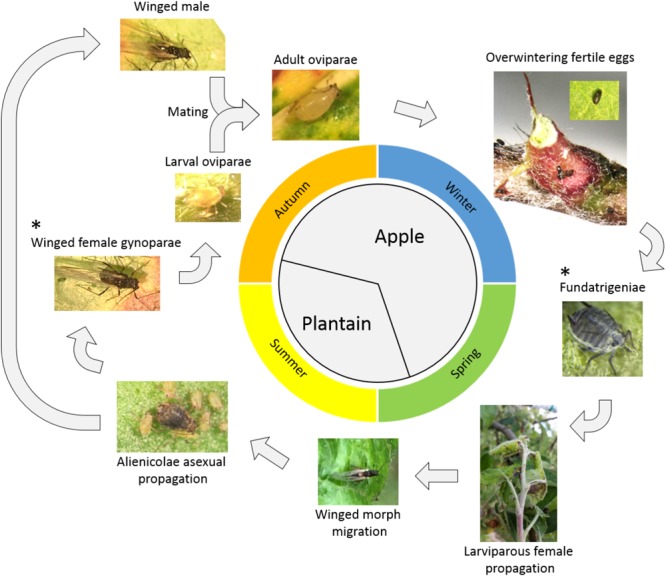
Life cycle of *Dysaphis plantaginea*. ^∗^ denotes the two different forms of aphid subjected to antibiosis and antixenosis experiments in our study.

*Dysaphis plantaginea* is one of the most important pests of apple orchards. This aphid causes leaf-rolling, shoot distortion and fruit deformation leading to unmarketable products (Wilkaniec, 1993). *Dysaphis plantaginea* has a holocyclic lifestyle with two successive host plants: apple and plantain (*Plantago lanceolata*) (Blommers et al., 2004) (Figure 1). Overwintering eggs deposited around the buds or in bark crevices of apple trees hatch in early spring, giving rise to fundatrices, further giving rise to several generations of wingless larviparous females, the fundatrigeniae, responsible for most of the damages. In early summer, winged females are produced, which migrate to their alternative host, plantain, and give rise to several generations of wingless larviparous females, the alenicolae. In early fall, the alienicolae give first birth to a winged generation of gynoparae, which migrate back to apple, giving birth to the sexual wingless females, the oviparae. Two to three weeks after gynoparae migration, alenicolae give birth on plantain to winged males, which migrate to apple, where mating with adult oviparae takes place, followed by oviposition. Two stages of the life cycle are particularly important as targets for control methods: (i) fundatrigeniae on apple during spring, because of their massive parthenogenetic reproduction, causing severe damage, and (ii) winged gynoparae females developing in the fall on plantain, because they migrate toward apple and obviously influence the number of overwintering eggs that will be layed.

Aphids use different sensory modalities to choose their host plants. Both visual and olfactory cues are involved in host finding over a distance. Aphids use colors, visual patterns, and contrast as well as volatiles emitted by plants to distinguish suitable from non-suitable host plants (Döring, 2014). Host acceptance after contact with a plant depends on additional cues: at close range, aphids evaluate host plants through surface cues, nutritional quality (i.e., gustatory stimuli) and accessibility of phloem (Pickett et al., 1992; Tjallingii, 2006).

In the present work, we demonstrate that ASM impacts several life traits of *Dysaphis plantaginea*. A transcriptomic analysis of ASM-treated apple tissues allowed us to fully explore the transcriptional reprogramming of apple. We identified two strongly up-regulated gene families coding for proteins with a potential to modify *Dysaphis plantaginea* biology and explored their role in resistance by antibiosis (affecting life traits) and antixenosis (affecting behavior): genes of the terpene-synthesis pathway, leading to modified volatile emission, and genes of the lectin family, which could interfere during aphid feeding.

## Materials and Methods

### Plant Material and Growth Conditions

All experiments were performed on apple (*Malus* × *domestica*) cv. Golden Delicious, chosen because of its susceptibility to *Dysaphis plantaginea* infestation (Robert et al., 2016). Seedlings from open pollination were used for gene expression, volatile organic compound (VOC) and protein profiling (four to six leaves stage, 5-week-old plants) and for the aphid choice tests (10–15 leaves stage, 12-week-old plants). Actively growing scions grafted on MM106 rootstocks (12-week-old plants) were used to study life traits (longevity, fecundity and larval development) and feeding behavior of aphids. Plants were grown under greenhouse conditions (natural photoperiod supplemented with artificial light if needed during a 16 h photophase, 23–25°C day, 17°C night).

### Compounds

To assess ASM action, plants were sprayed to runoff (with a pressurized hand sprayer) with the commercial product Bion^®^ 50WG (Syngenta, Basel, Switzerland; 50% of ASM) prepared in reverse osmosis water at a final concentration of 0.4 g l^-1^. The same water was used as control because the formulation blank is not available. To study the life traits and feeding behavior of aphids on apple scions, solutions were applied three times at 16, 9, and 2 or at 7, 5, and 3 days before infestation (dbi), respectively. For the other experiments, performed on seedlings, plants were sprayed once 3 days before sampling or infestation.

To evaluate the effect of (*E,E*)-α-farnesene (Bedoukian, Danbury, CT, United States) and (*E*)-β-caryophyllene (Sigma-Aldrich, St. Louis, MO, United States) in the olfactometer system, decadic dilutions ranging from 10 μg μl^-1^ to 1 ng μl^-1^ were prepared in 100% hexane (Sigma-Aldrich, St. Louis, MO, United States). Hexane was used as a control.

### Aphid Rearing

Fundatrices of *Dysaphis plantaginea* were collected in early spring on apple trees in Angers (France) and placed on apple scions for the production of fundatrigeniae. Adult individuals were further propagated on apple scions without differentiating individual clones. This rearing was carried out in a climatic chamber under long day conditions: day (16 h, 22°C, 80% RH) and night (8 h, 18°C, 90% RH), with daylight fluorescent tubes. For the production of gynoparae, colonies of clonal alienicolae (kindly provided by M. Siegwart, Avignon, France) were reared on plantain in a climatic chamber under short day conditions: day (13 h, 19°C, 70% RH) and night (11 h, 18°C, 80% RH). When necessary, aphids were gently manipulated with a paintbrush. These conditions allowed parthenogenetic reproduction with development of mature females within 10 days (fundatrigeniae) or 15 days (gynoparae).

### Evaluation of Life Traits

To assess the lifespan and fecundity of adult *Dysaphis plantaginea* fundatrigeniae, apple scions, either control- (*n* = 39) or ASM-treated (*n* = 35), were each infested with one freshly emerged adult from a synchronized rearing, and maintained under the same environmental conditions as used for the rearing. At each of the eight different recording dates (every 3 or 4 days during 26 days), the survival of the deposited individuals was verified (longevity rate), and newly born nymphs were counted and subsequently removed (fecundity).

To assess the mortality and development duration of the first generation of nymphs produced on either control- (*n* = 39) or ASM-treated (*n* = 37) apple scions, two to three adult fundatrigeniae were deposited per plant, allowed to give birth for 24 h and were then removed. Nymphs were counted at the start of the experiment (*n* = 439 for control plants and *n* = 508 for ASM plants) and larval development (viability over time and time to reach the adult stage) was monitored 12, 15, 18, and 21 days after infestation, with newly emerged mature females removed to avoid production of second-generation nymphs.

In both types of experiments, aphids were prevented to move from one plant to the other by separating each pot from the other in order to avoid leaf contact, and by positioning pots on stilts in a water-filled bench in order to avoid mobility over the ground.

### Study of the Feeding Behavior

Feeding behavior was assessed using the electrical penetration graph (EPG) DC-system (EPG systems, Wageningen, Netherlands, Tjallingii, 1985). Each adult fundatrigeniae was placed on a youngest expanded leaf of an individual apple scion and dorsally tethered with a thin gold wire (diam. = 20 μm) using conductive silver glue. The reference electrode was inserted into the soil of the potted plant. Each aphid-plant system (*n* = 23 for control and *n* = 27 for ASM) was placed in a Faraday cage at 20 ± 1°C, 35–45% RH, under natural light conditions. Acquisition and analysis of the EPG waveforms were carried out with the EPG Stylet+ daq and EPG Stylet+ ana softwares, respectively. Signals were recorded during the photophase for 8 h and analyzed according to Tjallingii and Esch Hogen (1993). The following variables were deduced from the temporal sequence of waveforms recorded per aphid, i.e., number, average time, total duration of non-probing and waveforms C (stylet pathway in epidermis and mesophyll), E1 (phloem sieve salivation), E2 (phloem sap ingestion), F (stylet derailment) and G (xylem ingestion) as well as time from start to the first C, E1 and E2 events and their respective duration, and time between first E1 and first E2.

### Plant Choice Test

Host plant selection by aphids was evaluated on 12-week-old apple seedlings for adult fundatrigeniae and gynoparae, using overnight-starved individuals. For fundatrigeniae, five adults raised on untreated apples were placed on each plant, either control- or ASM treated, and stems of each pair of differentially treated plants were attached to each other to allow movement of aphids between the two plants. The experiment was repeated on five pairs of plants. For gynoparae, emerged on plantain, ready to migrate on apple, 20 individuals were placed in an open Petri dish in a cage (50 cm × 50 cm × 50 cm) containing two control- and two ASM-treated plants. The number of aphids on each plant was recorded 24 h later. The experiment was repeated two times with two cages (*n* = 4). Both experiments were performed under natural light under the following conditions: day 15 h; 23°C; night 9 h; 20°C; 35–45% RH.

### VOC Capture and Identification

Volatile organic compound emitted by 5-week-old apple seedlings were collected *in situ* using the dynamic headspace technique (Cornille et al., 2012; Dormont et al., 2013). The experiments took place during the morning in early spring under natural light and with the temperature stabilized to 23–25°C. Batches of 20 similarly treated plants were enclosed together in a polyethylene terephthalate bag (Nalophan). A batch of ASM-treated plantless pots was also similarly prepared in order to verify the lack of emission of VOC from bags, pots, earth and ASM. After 30 min of these confined conditions allowing the accumulation of scent, the headspace was then vacuum extracted (flow rate of outcoming air: 200 ml min^-1^) for 10 min through adsorbent traps, Chromatoprobe^®^ quartz microvials of Varian Inc. (length: 15 mm; inner diameter: 2 mm) previously cut closed-end and containing 1.5 mg of Carbotrap^®^ 20–40 mesh and 1.5 mg of Tenax 60–80 mesh (Sigma-Aldrich, Munich, Germany). Adsorbent traps were previously loaded with a known amount of biphenyl (11.1 ng – Sigma-Aldrich Merck > 99%) as an internal standard. This accumulation/extracting cycle was repeated six times to increase the quantity of volatiles trapped on the filter. Experiments were repeated six times for control- and ASM-treated plants, as well as for ASM-treated plantless pots and performed in parallel. Three supplemental repeats were performed under the same conditions for ASM-treated plants. Trapped odor samples were stored at -18°C until analysis.

Samples were analyzed at the “Platform for Chemical Analyses in Ecology” (PACE), technical facilities of the LabEx CeMEB (Centre Méditerranéen pour l’Environnement et la Biodiversité, Montpellier, France). Samples were desorbed 6 min in a Gerstel Thermal Desorption Unit (by a Gerstel MPS autosampler, Gerstel GmbH & Co. KG, Mülheim an der Ruhr, Germany) and then injected via the Gerstel Cooled Injection System (-80°C then 250°C) with a split ratio of 4:1 into a coupled Gas Chromatography-Mass Spectrometry (GC-MS) system (Thermo Trace 1310-ISQ QD Single Quadrupole, Thermo Fisher Scientific Inc. – Bremen, Germany) equipped with a 30 m DB-5 MS column (methyl siloxane, 0.25 μm film thickness, 250 μm ID, Macherey-Nagel, Düren, Germany) and helium as the carrier gas (1 ml min^-1^). Ionization was by electron impact (70 eV, source temperature 200°C). The column temperature was held at 40°C for 3 min with a following program of 5°C min^-1^ up to 200°C, then 10°C min^-1^ up to 250°C, kept for 2 min. The overall analysis lasted 48 min. Calibration samples were used to quantify the two sesquiterpenes (*E*)-β-caryophyllene and (*E,E*)-α-farnesene.

All GC-MS data were processed in a forked version of MZmine 2.18 (Pluskal et al., 2010) adapted to GC data processing (customized software available on demand), using the same automated protocol ensuring the consistency of peak integration. Following normalization of the dataset, peaks were detected by local minima chromatogram deconvolution and aligned across samples based on mass spectra and retention times.

Identification of (*E*)-β-caryophyllene was done by mass spectra comparison to the commercial standard and libraries: Wiley registry of Mass Spectral Data – 9th ed., NIST MS Database 2011 and Adams (2007). (*E,E*)-α-farnesene and germacrene-D identified by comparing the mass spectra to the same libraries and manual confirmation of the identification by comparing Kovats indices in literature (Adams, 2007).

### Olfactometer Test

Gynoparae were starved overnight in a Petri dish containing a moist filter paper disk. A straight glass tube with a hole in the center was used as an olfactometer. Ten microliters of an odor solution, resulting in compound amounts ranging from 10 ng to 100 μg, were applied on a piece of filter paper introduced in a 3 cm long glass tube and connected to one end of the olfactometer with a piece of silicon tubing. A glass tube containing a filter paper with the same amount of solvent (hexane) was connected to the other end of the olfactometer. Each stimulation tube was used for three consecutive experiments and the olfactometer was turned 180° after each experiment. The olfactometer was placed in a cardboard box lined with white filter paper and illuminated from above with two 18 W daylight tubes to avoid any phototactic bias of aphid behavior. Experiments were done in the morning during the first 6 h of the photophase at a temperature between 20 and 23°C and 35–45% relative humidity. A single aphid was introduced into the central hole and an airflow of 150 ml min^-1^ through both halves of the olfactometer was generated by a sucking PTFE membrane pump (KNF Neuberger, France). The contaminated air was evacuated through the window. Each aphid was observed during 10 min and the time spent on each side of the olfactometer was recorded. At least 30 aphids were tested for each dose of each tested compound.

### Microarray Analysis

Gene expression profiling was performed using apple chip AryANE V1.0 containing 135,000 oligoprobes of which 126,022 correspond to predicted coding genes and microRNA precursors of apple var. Golden Delicious (Velasco et al., 2010; Celton et al., 2014): 63011 predicted as complementary with coding sequences (CDS) and 63011 predicted as complementary with antisense. Each sample corresponds to a bulk of the youngest expanded leaf from five plants. Hundred mg of youngest expanded leaf tissues were ground (Retsch, Haan, Germany), and RNA was extracted with the Macherey-Nagel Nucleospin RNA Plant Kit (Macherey-Nagel, Düren, Germany) and by adding 2% PVP-40 in the extraction buffer. The quality of RNA was verified in 1.2% agarose gel before amplification steps. Targets were prepared, labeled, hybridized on the slide and scanned according to Celton et al. (2014).

### *Amaranthin-Like Lectin* Gene Identification and Protein Modeling

Functional annotation of amaranthin-like lectin protein sequences was performed with Interproscan version 5 (Jones et al., 2014) using corresponding protein sequences retrieved from the Genome Database for *Rosaceae* as queries^1^. Homology search by tblastn was performed using these protein sequences as queries and the apple GDDH13 v1.1 genome sequence as database^2^. Protein modeling was performed with Phyre2 web server (Kelly et al., 2015) and the output pdb file was further modeled for homo-oligomer structures search using GalaxyGemini web server (Lee et al., 2013). Protein model visualization and molecular docking experiments were performed with USCF Chimera v1.12 software (Pettersen et al., 2004) implemented with Autodock Vina (Trott and Olson, 2010).

### Protein Extraction

Apple proteins were extracted as recommended by Wang et al. (2006b) with some specificities: 200 mg of young growing tissues were ground into a fine powder in the presence of 10 mg of polyvinylpolypyrrolidone (PVPP). In the last step, proteins were dissolved in PBS buffer (2.4 mM KH_2_PO_4_, 140 mM NaCl, 7.4 mM Na_2_HPO_4_, pH 7.2).

### SDS-PAGE and Western Blot-Immunodetection

The samples were mixed with loading buffer (0.312 M TRIS, 50% glycerol, 10% SDS, 0.05% bromophenol blue, 25% β-mercaptoethanol) (4:1 v:v) and heated at 80°C for 5 min. The samples were separated on 14% SDS-PAGE (120 V, 1 h), stained with Coomassie Brilliant blue (0.25% Coomassie Brilliant blue, 50% methanol, 10% acetic acid) or electroblotted (60 mA/gel, 1 h, <30 V) onto 0.45 μm polyvinylidene difluoride (PVDF) membranes (immobilon-P, Milli- pore Corp., Bedford, MA, United States). Coomassie gels were destained by repeated washings in ethanol:acetic acid solutions. After protein transfer, the PVDF membrane was immersed in a blocking solution (PBS, 0.05% Tween, 10% milk powder) overnight at 4°C and successively washed three times in PBS buffer, incubated 1.5 h in PBS solution containing 1:1000 of two custom-made rabbit anti-MdAGG antibodies (Eurogentec EGT GROUP, Seraing, Belgium), and washed three times in Tris-NaCl solution (100 mM Tris, 100 mM NaCl, pH 7.5). Finally, the membrane was incubated in Tris-NaCl solution with 1:200000 alkaline phosphatase-conjugated goat anti-rabbit antibody (1h30) and immunodetection was performed with a NBT/BCIP solution (100 mM Tris, 100 mM NaCl, 10.7 mM MgCl_2_, 0.01% [10 mM NBT, 70% *N*-*N*-dimethylformamide], 0.0075% [135 mM BCIP, 100% *N*-*N*-dimethylformamide], pH 9.6). The reaction was stopped by adding 0.2 M HCl.

### Statistical Analysis

All statistical analyses were performed using the R language (R Development Core Team, 2016, R version 3.3.1).

The effect of ASM treatment on adult fundatrigeniae fecundity between each observation date was globally assessed with Generalized Linear Mixed Models using the packages LM4 and Car. The explanatory variables were: the treatment (control/ASM) and count dates, whereas the plant was included as random effect. The response variables were counts of individuals; so a Poisson error structure was used for the analyses. Model assumption was checked using the package RVAideMemoire (Hervé, 2014). Adult and larval survival experiments, and the time needed to reach the adult stage were analyzed using the Cox proportional hazard model (package survival) to globally assess the effect of ASM-treatment on these life traits. Olfactometer data were analyzed statistically with a paired *T*-test and plant choice tests as well as EPG data were analyzed with a Mann and Whitney test. Statistical values for the analysis of life traits, olfactometer experiments, plant choice and EPG are provided in Supplementary Table S1.

For microarray, raw data analysis was performed according to Celton et al. (2014). A probe was flagged as differentially expressed if the log_2_ ratio was ≤-0.5 or ≥0.5 and if the corrected *p*-value was ≤0.05. Enrichment tests of Gene Ontology terms (GO) were performed by comparing effectives to a hypergeometric distribution using GO.db package and phyper function.

## Results

### *Dysaphis plantaginea* Life Traits Are Affected in ASM-Treated Plants

Once verified that ASM has no direct detrimental effect on *Dysaphis plantaginea* fundatrigeniae (Supplementary Table S2), several aphid life traits, i.e., adult lifespan, fecundity per female, larval lifespan and development kinetics, were analyzed to determine if ASM induces resistance by antibiosis. Global statistical analysis showed that all life traits were significantly affected by ASM treatment (Supplementary Table S1).

**FIGURE 2:**
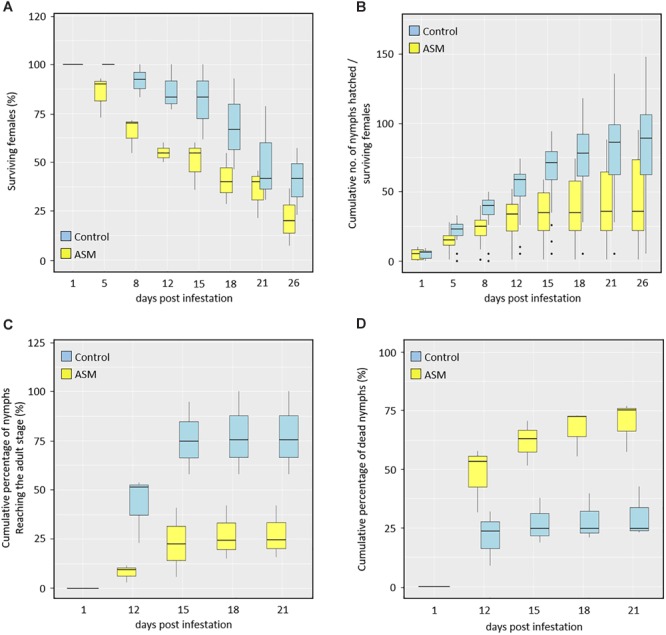
Effect of ASM treatments on *Dysaphis plantaginea* fundatrigeniae life traits on apple. ASM or control was applied three times (at 16, 9, 2 dbi) before infestation with one **(A,B)** or two/three **(C,D)** mature females per apple scion. Box plots representing **(A)** longevity of surviving females on a total of 39 plants for control and 35 for ASM treatment, **(B)** cumulative number of nymphs produced per surviving females produced on the same plants as in **(A)**. **(C,D)** Box plots representing larval evolution of nymphs produced from two females deposited per control-treated plant (*n* = 39 plants) and from three females deposited per ASM-treated plant (*n* = 37 plants): **(C)** cumulative percentage of nymphs evolving over time into adult individuals and **(D)** cumulative percentage of nymphs dying over time. The box plot shows the median (horizontal line), the second and third quartile (box), the first and fourth quartile (whisker) as well as outlier data (dots).

Considering the infesting fundatrigeniae lifespan (Figure 2A), no mortality was observed 1 day after infestation, regardless of the treatment. Starting from day 5, adult individuals placed on ASM-treated plants began to die whereas no mortality was observed for control-treated plants. The proportion of dead aphids found on ASM-treated plants increased more rapidly than on control plants. Regarding fecundity of the surviving aphids (Figure 2B), again no difference was observed between *Dysaphis plantaginea* infesting ASM- or control-treated plants at the beginning of the experiment, but from that time point on, fecundity was lower for aphids placed on ASM-treated plants.

In a separate experiment, the development of fundatrigeniae nymphs either produced on control- or ASM-treated plants was followed up from day 12 to day 21 after infestation (Figures 2C,D). On control plants, nymphs reached the adult stage faster and to a higher proportion than those placed on ASM-treated plants (Figure 2C). Larval mortality over time was also lower on control-treated plants than on ASM-treated plants (Figure 2D).

Altogether, these results show that ASM treatment induces resistance by antibiosis, which significantly affects several important life traits of *Dysaphis plantaginea* fundatrigeniae.

### *Dysaphis plantaginea* Choice and Feeding Behavior Is Affected by ASM Treatment

Behavioral studies were performed in order to determine if ASM treatment induces resistance to *Dysaphis plantaginea* in apple by antixenosis. Choice tests for both adult fundatrigeniae and gynoparae revealed a clear preference for control-treated plants. Whatever the morph considered, the proportion of aphids was significantly higher on control- than on ASM-treated plants (Table 1). This was especially true for gynoparae, which were found on control plants at over 90%.

**Table 1:** Test of choice of *Dysaphis plantaginea* between control- and ASM-treated plants.

	Fundatrigeniae	Gynoparae
**Control**	73.64 ± 2.95	93.75 ± 3.61
**ASM**	26.36 ± 2.95 *	6.25 ± 3.61 *

*Mean percentage from five independent experiments (10 adult fundatrigeniae equally distributed between control and ASM-treated plants) or from 2 cages × 2 independent experiments (20 gynoparae released in a Petri dish situated between the four plants/cage). ^∗^Significant differences (Mann–Whitney test; *p*-value < 0.05).*

**FIGURE 3:**
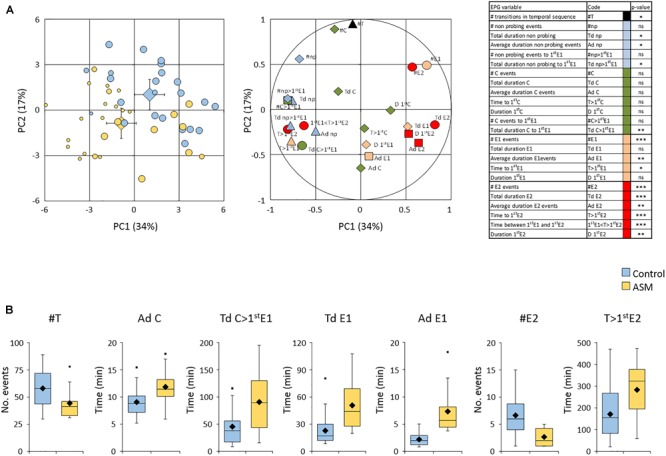
Effect of ASM treatments on *Dysaphis plantaginea* fundatrigeniae probing behavior. ASM or control was applied three times at 7, 5, 3 dbi. **(A)** Principal component analysis of EPG data (*n* = 23 aphids on control-treated plants and *n* = 27 on ASM-treated plants). Left panel: scatterplots of individual aphids on PC1 vs. PC2. The size of circles indicate on the number of phloem sap ingestion E2 events recorded for each aphid: zero (small circles), only one (medium circles), more than one (big circles). Large diamonds indicate barycenters with confidence intervals of each batch of aphids. Right panel: correlation of EPG variables on PC1 vs. PC2. Variables with significant p-value between the two batches of aphids are indicated with symbols (diamonds: not significant; triangle: *p* < 0.05; square: *p* < 0.01; circle: *p* < 0.001). Table: codes of EPG variables with *p*-values resulting from the comparison between the two batches of aphids (Mann and Whitney; ns: not significant; ^∗^*p* < 0.05; ^∗∗^*p* < 0.01; ^∗∗∗^*p* < 0.001). **(B)** Analyses of aphids successfully reaching at least one E2 event (*n* = 22 aphids on control-treated plants and *n* = 12 aphids on ASM-treated plants). Focus on significant EPG variables with box plots representing distributions of aphids. Medians, means and outliers are indicated with horizontal lines, diamonds, and squares, respectively.

Adult fundatrigeniae feeding behavior was then monitored using the EPG technique. A total of 23 and 27 aphids positioned on control- and ASM-treated plants, respectively, were recorded for 8 h. A principal component analysis was performed on the data set with variables deduced from the temporal sequence of waveforms recorded per aphid (Figure 3A). The waveforms F (stylet derailment) and G (xylem ingestion) were not retained because of the high number of aphids not displaying these two non-phloem behaviors (F: 10 and 13 aphids; G: 17 and 26 aphids on control- and ASM-treated plants, respectively). The scatter plot of individuals revealed a separation between the two populations of aphids with, however, some overlapping, but suggested that aphids were affected in their probing activities. Both principal components explained differences in aphid behaviors.

Amongst the variables contributing the most to these components, some discriminated the two batches of aphids significantly (see Figure 3A and Supplementary Table S1). Aphids exhibited a lower global activity on ASM-treated plants as emphasized by a lower number of transitions in the temporal sequence of waveform phases (black symbol, Figure 3A). All variables related to non-probing were increased (blue symbols, Figure 3A), with a significantly longer period without stylet penetration. The non-phloem probing events C (green symbols, Figure 3A) were not affected on the whole temporal sequence. However, a significantly longer time of these intracellular punctures in non-phloem tissue was necessary before reaching the first event of salivation after sieve element puncture (E1). Considering the total recording time, or specifically the time necessary to reach the first phloem sap ingestion E2 (if any, see below), less events of salivation E1 were observed, but each E1 event lasted longer on average (orange symbols, Figure 3A). With regard to E2 events, only 12 out of 27 aphids (44%) were able to have at least one sap ingestion event on ASM-treated plants (while 85% exhibited E1 activities), vs. 22 out of 23 (96%) on water-treated ones (with 100% exhibiting E1 activities) (medium and big circles on the scatterplot of individuals, Figure 3A). All variables depicting E2 events (red symbols, Figure 3A) were significantly affected for aphids recorded on ASM-treated plants, but the high number of aphids unsuccessful in reaching any sap ingestion event could introduce a bias in statistical analyses. We therefore re-conducted the analyses on aphids accomplishing this feeding event (Figure 3B). Seven variables were still significantly different between aphids probing on ASM-treated and water-treated plants: the number of transitions in the temporal sequence (an average of 44 vs. 58 transitions, respectively), the average duration of C events (11.8 vs. 9.0 min per event), the total duration of C to reach the first E1 event (90.8 vs. 45.4 min), the total and average durations of E1 events (7.3 vs. 2.2 min per event), the number of E2 events (2.6 vs. 6.6 events) and the time to reach the first E2 (283 vs. 172 min). Altogether, these results show that fundatrigeniae exhibit a reduced stylet-activity on ASM-treated plants, with difficulties to locate the sieve elements and to accomplish sap ingestion.

### ASM Treatment Induces Defense-Oriented Reprogramming of Apple Transcriptome

**FIGURE 4:**
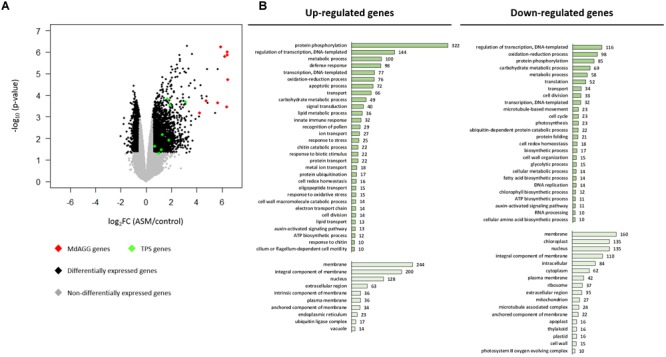
Apple leaf transcriptome remodeling by ASM treatment. **(A)** Volcano plot representing gene expression modulation 3 days after ASM treatment. Each scattered point represents a single gene: the *x* coordinates corresponds to the log_2_ fold change (log_2_FC) of the expression ratio between ASM- and control-treated plants; the *y* coordinates correspond to –log_10_ of the *p*-value (*n* = 4 samples per treatment from four independent experiments). Genes involved in the terpene and agglutinin pathways are highlighted in green and red, respectively. **(B)** Selected results (gene number > 10 and hypergeometric test ≤ 0.05) of Gene Ontology terms over-representation analysis for up- (left panels) and down-regulated genes (right panels). Dark green barplots represent Biological Process (BP) and light green barplots Cellular Component (CC). *X*-axis corresponds to the number of genes differentially expressed included in each category.

To gain insight into the ASM-induced mechanisms affecting *Dysaphis plantaginea*, a microarray analysis was performed 3 days after treatment. Genes were considered differentially expressed upon ASM treatment for log_2_ fold changes |log_2_FC|≥ 0.5 and *p*-values ≤ 0.05. Using these criteria, 4,327 genes were considered as differentially expressed in response to ASM; 2,398 genes being up-regulated and 1,929 being down-regulated upon treatment. Though the proportion is relatively similar in genes up- or down-regulated, we observed that induction levels were stronger than repression levels (Figure 4A). To determine which particular classes of genes were regulated by ASM, we performed GO analysis on up- and down-regulated genes separately (Figure 4B and see Supplementary Table S3 for selected genes). Strikingly, we observed that up-regulated genes are associated with biological processes involved in plant immunity (GO “defense response,” “apoptotic process,” “innate immune response,” “response to stress,” “response to biotic stimulus,” “chitin catabolic process” and “response to chitin”) while down-regulated genes are associated to primary functions (“translation,” “cell division,” “cell cycle,” “photosynthesis,” and “protein folding”). The most important class overrepresented in up-regulated genes is “protein phosphorylation” (*n* = 322), including receptors involved in pathogen perception like FLS2, WAK, and CRK and genes involved in signal transduction associated with defense reaction like MAPK, calmodulin and CBL (also present in “signal transduction” category). Numerous up-regulated genes are also involved in regulation of transcription (“regulation of transcription, DNA templated” and “transcription, DNA templated” also present in “nucleus” GO categories). The most important family present in this category corresponds to WRKY transcription factor (32/144 genes in “regulation of transcription, DNA templated”), well-known to be involved in transcriptional reprogramming associated with the plant immune response (Rushton et al., 2010). Many genes encoding PR-proteins (PR1, PR2, PR4, PR5, PR8, and PR14) were also up-regulated and present in the categories: “defense response,” “protein phosphorylation,” “carbohydrate metabolic process,” “lipid transport.” We also found eight genes involved in terpene biosynthesis included in “metabolic process” (8/100) GO (Figures 4A,B).

For down-regulated genes, GO analysis revealed that numerous genes are included under the GO Cellular Component term “chloroplast” (*n* = 135), which is absent for up-regulated genes. Some of these are involved in photosynthesis (9/135), Calvin cycle (8/135) (also present in “carbohydrate metabolic process”) and starch metabolism (6/135). Three Cellular Component GO terms are associated with intercellular compartment (“extracellular region,” “apoplast,” and “cell wall”). These categories include some genes involved in cell wall polysaccharide remodeling. Numerous down-regulated genes are involved in “translation” (*n* = 52), “cell division” (*n* = 33) and “cell cycle” (*n* = 23). Categories “regulation of transcription, DNA templated” (*n* = 116) and “transcription, DNA templated” (*n* = 32) encompass genes acting in the auxin-signaling pathway and in transcription initiation (Figure 4B).

Altogether, this analysis showed that ASM treatment triggers an important transcriptome reprogramming in apple. Mechanisms involved in perception, transduction and signaling associated with SA-dependant immunity were up-regulated, whereas mechanisms involved in cellular division, translation, cell wall remodeling and photosynthesis were in contrast repressed by ASM. Among all the differentially expressed genes, those encoding terpene synthase might be related to aphid resistance by antixenosis, because terpene volatiles are known to be repellent for many herbivorous insects (Unsicker et al., 2009). However, no obvious gene regulation related to antibiosis was detected, and unfortunately most of the genes overexpressed with a log_2_FC > 4 were not functionally annotated. Interproscan analysis of their predicted protein sequence showed that nine of these genes harbored the Pfam domain PF07468, termed agglutinin or amaranthin domain, because initially described for the *Amaranthus caudatus* amaranthin lectin (Figure 4A), a protein which is toxic to aphids (Wu et al., 2006; Xin et al., 2011).

### Identification and Characterization of Apple Amaranthin-Like Lectin Encoding Genes Highly Up-Regulated by ASM

We took advantage of the high-quality genome assembly of the Golden Delicious Double Haploid 13 (GDDH13, Daccord et al., 2017) and performed a tblastn search against the translated nucleotide sequence of the GDDH13 genome using protein sequences of these previously identified agglutinins and those from other plant species as queries. Each detected locus was manually annotated to decipher gene function. This led to the identification of 27 putative genes encoding proteins with either one or two agglutinin domains. Among these, 14 had been automatically annotated as genes in the apple genome version of 2010, but only three were in the GDDH13 version of 2017. Deepening the analysis of those sequences led to the identification of 18 putative genes encoding proteins with one agglutinin domain and predicted molecular weights ranging from 18.9 to 20.6 kDa (termed *MdAGG1* to *MdAGG18*) and nine putative genes encoding proteins with two agglutinin domains and predicted molecular weights ranging from 34.5 to 59.7 kDa (termed *MdDiAGG1* to *MdDiAGG9*, Table 2). *MdAGG7, MdDiAGG8* and *MdDiAGG9* were defined as pseudogenes in the GDDH13 genome because their CDS are disrupted by frameshifts and/or stop codons. Interproscan analysis of the complete protein sequences showed that MdDiAGG5, 6 and 7 harbored at their C-terminal end the SCOP domain SSF56973 corresponding to an aerolisin/ETX pore-forming domain. Strikingly, *MdAGG1* to *MdAGG15* are clustered in a 250 kb region on chromosome 10 and have a high percentage of sequence identity, whereas all other genes are scattered throughout the apple genome (Supplementary Figures S1, S2). Expression data showed that the genes encoding mono-agglutinin were highly induced by ASM treatment, whereas those encoding di-agglutinin were not differentially expressed upon ASM treatment, except *MdDiAGG4* for which one probe showed weak but significant repression. We therefore only characterized MdAGGs.

**Table 2:** Characteristics of 17 *MdAGG* genes and 7 *MdDiAGG* genes in *Malus* × *domestica* ‘Golden Delicious’ double haploid 13.

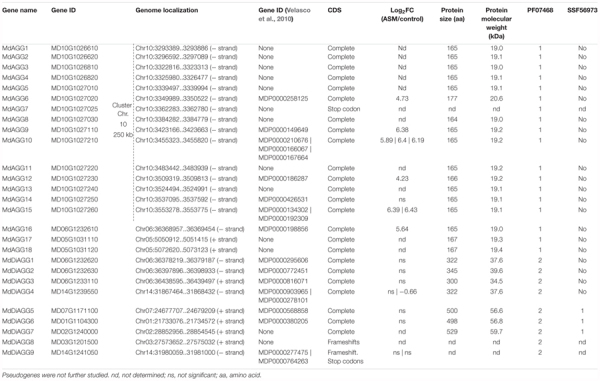

**FIGURE 5:**
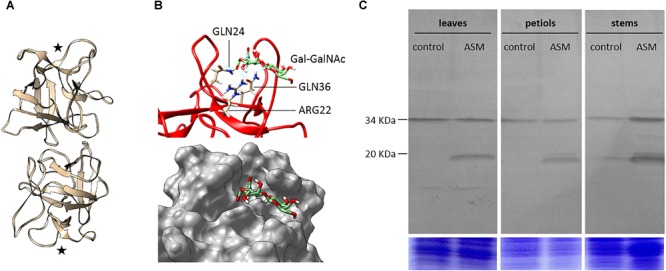
Molecular modeling of MdAGG and accumulation in ASM-treated apple tissues. **(A)** Ribbon diagram of the dimeric structure of MdAGG10. Both monomers consist of a single agglutinin domain and each one contains a putative carbohydrate-binding site (stars). **(B)** Focus on the carbohydrate-binding site predicted to anchor Gal-GalNAc disaccharide (green sticks). Amino acid residues GLN24, ARG22, and GLN36 predicted to interact with GalNAc are represented as sticks (upper panel). Surface view of the predicted binding pocket anchoring Gal-GalNAc (lower panel). **(C)** Western blotting analysis of MdAGGs from leaves, petioles and stems apple proteins extracted 3 days after control or ASM treatment (upper panels). The total amount of Coomassie-stained proteins in each lane from a gel with duplicated samples serves as a loading control (lower panels). Data are representative of two independent experiments.

The crystal structure of *A. caudatus* amaranthin (PDB: 1jlx; GenBank: AAL05954; further referred as AcaAGG), a protein composed of two adjacent agglutinin domains associated head to tail as a homodimer, allows us to propose a predicted consensus 3D model of the MdAGG proteins. Protein sequence alignment of AcaAGG and all MdAGGs showed that the latter share from 32.2 to 34% identity in the N-terminal agglutinin domain of AcaAGG. Interestingly, most of the amino acids corresponding to the β-sheets of AcaAGG were conserved in all MdAGGs (Supplementary Figure S3). Modeling of MdAGG10 (one of the most up-regulated genes after ASM treatment) resulted in a predictive homodimer composed of two β-trefoil domains associated tail-to-tail, reminiscent of the crystal structure of the *Boletus edulis* lectin (PDB: 4i4o; Figure 5A). Molecular docking was performed to determine if the cryptic T-antigen disaccharide galactose-*N*-acetylgalactosamine (Gal-GalNAc), crystalized with AcaAGG is predicted to bind to MdAGG10. Albeit the carbohydrate-binding site at the interface of two adjacent agglutinin domains of AcaAGG is not predicted to be restored in our protein model, MdAGG10 was predicted to bind Gal-GalNAc through six hydrogen bonds located in a pocket at each extremity of the homodimer (Figure 5B). Interestingly, the three amino acids involved in this putative interaction are conserved in all MdAGGs and in AcaAGG. However, they do not correspond to the amino acids of AcaAGG that bind Gal-GalNAc (Supplementary Figure S3). Using this model, we defined two oligopeptides conserved in most of the MdAGGs and present at their predicted surface, in order to synthetize rabbit antibodies raised against MdAGG proteins. Western blot analysis of proteins extracted from apple leaf, petiole and stem tissues showed that these antibodies detected a single protein of ≈34 kDa and several proteins with molecular weights of ≈20 kDa. The 34 kDa protein was detected whatever the treatment and could correspond to MdDiAGG3 because of sequence similarities in the regions that were used to design the synthetic peptide (Supplementary Figure S3). On the other hand, the 20 kDa proteins were only detected after ASM treatment and therefore correspond to MdAGGs.

### (*E,E*)-α-Farnesene Is Produced in Response to ASM Treatment and Is Repellent for *Dysaphis plantaginea*

**FIGURE 6:**
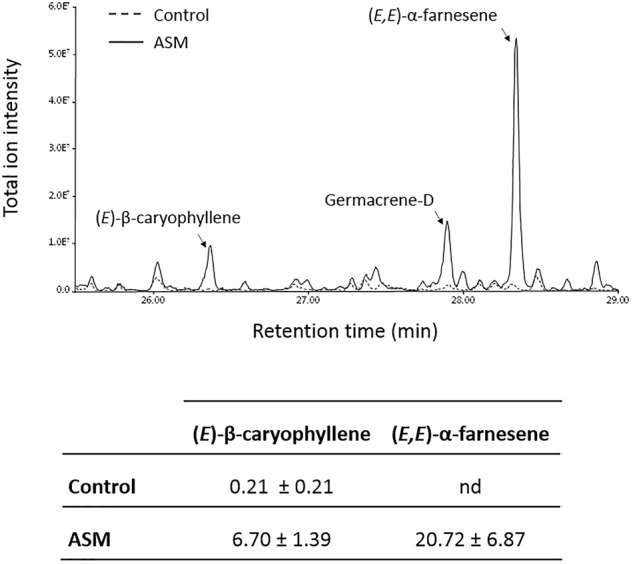
VOC emitted by ASM-treated plants. Representative chromatogram obtained with control and ASM-treated plants 3 days after treatment. Means (±SEM) of (*E*)-β-caryophyllene and (*E,E*)-α-farnesene amounts (pg h^-1^) from six (control) and nine replicates (ASM) of 20 plants from one experiment.

The transcriptome analysis revealed several terpene synthase-encoding genes induced after ASM treatment. Among these, MDP0000199152 has been shown to produce mainly (*E,E*)-α-farnesene, and MDP0000203143 produces (*E*)-β-caryophyllene and germacrene-D (Nieuwenhuizen et al., 2013), all three VOCs of the sesquiterpene family which can influence plant–insect relationships. Dynamic headspace trapping and gas chromatography-mass spectrometry (GC-MS) were performed in order to determine if ASM treatment leads to emission of these VOCs by apple plants. ASM-treated plants emitted more (*E,E*)-α-farnesene, (*E*)-β-caryophyllene and germacrene-D than control-treated plants (Figure 6). All three compounds were below the detection threshold for ASM-treated plantless pots, showing that when detected, these VOCs were emitted by plants (data not shown). Using commercially available (*E*)-β-caryophyllene and (*E,E*)-α-farnesene as standards allowed us to quantify their emission in response to ASM treatment (Figure 6). The former was present in very low quantity in control plants (0.21 pg h^-1^) and its production was enhanced 32-fold in response to ASM treatment (6.70 pg h^-1^). (*E,E*)-α-farnesene was below the detection threshold in control conditions whereas 20.72 pg h^-1^ were quantified after ASM treatment. These results show that ASM treatment modifies the VOC blend emitted by apple plants, leading in particular to the production of sesquiterpene volatiles, which are present in very low amounts or absent from the VOC blend of control plants.

**FIGURE 7:**
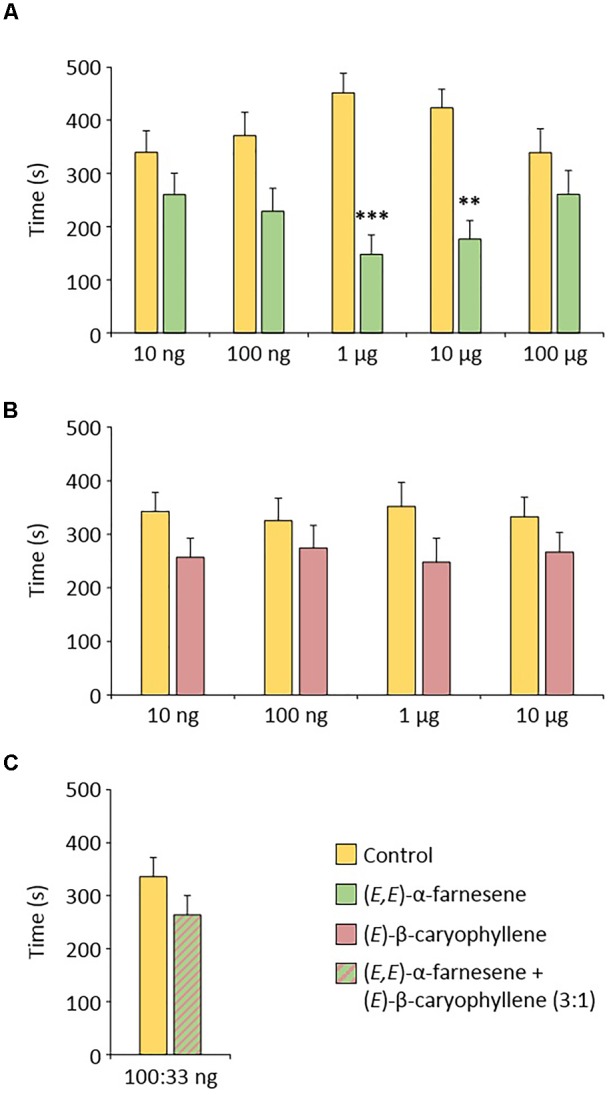
Behavioral effect of **(A)** (*E,E*)-α-farnesene, **(B)** (*E*)-β-caryophyllene and **(C)** the 3:1 (*E,E*)-α-farnesene/(*E*)-β-caryophyllene mixture on *Dysaphis plantaginea* gynoparae. Means (±SEM) from *n* = 30–33 aphids. Significant differences were calculated by a paired *T*-test (^∗∗^*p* < 0.01; ^∗∗∗^*p* < 0.001).

In order to determine if the compounds emitted by apple seedlings following the application of ASM can participate in the resistance phenomenon by antixenosis observed in these plants an olfactometric analysis was carried out to reveal a potential repellent effect of two identified compounds, commercially available, (*E,E*)-α-farnesene and (*E*)-β-caryophyllene. Gynoparae were used in these experiments, because they are likely to use VOC to choose apple plants during migration. Because repellent effects are dose-dependent, we tested a range of five doses for (*E,E*)-α-farnesene and four doses for (*E*)-β-caryophyllene. *Dysaphis plantaginea* gynoparae spent significantly more time in the hexane branch of the olfactometer than in the VOC branch for two doses of (*E,E*)-α-farnesene (1 and 10 μg). They were not significantly repelled at lower and higher doses (Figure 7A). (*E*)-β-Caryophyllene did not have a significant repellent effect on gynoparae (Figure 7B) and adding 33 ng of (*E*)-β-caryophyllene to a sub-threshold dose of (*E,E*)-α-farnesene (100 ng), did not provoke a significant repellent effect either (Figure 7C).

## Discussion

By showing that ASM, a functional homolog of SA, triggers resistance to *Dysaphis plantaginea* in apple, we were able to demonstrate that the SA signaling pathway is important for setting up resistance to this particular aphid. The gene expression profiling clearly demonstrated that ASM treatment is reprogramming the apple transcriptome toward defense mechanisms, and inhibits the expression of photosynthesis- and growth-associated genes. This transcriptomic remodeling has been commonly observed in plants subjected to pathogen attack or in response to plant resistance inducers (Appel et al., 2014; Zhou et al., 2015; Landi et al., 2017). Although no plant growth limitation was observed during the timeframe of our experiments (data not shown), potential long-term effects on apple trees in orchards have to be considered in the future.

Two complementary mechanisms were shown to be involved in aphid-resistance induced by ASM: antibiosis and antixenosis. Whatever the aphid morph considered, antixenosis has been in particular demonstrated by our choice tests, and more pronounced for gynoparae. The winged morph of aphids, essential for colonization of new habitats, is indeed usually more sensitive to antixenosis mechanisms (Morkunas et al., 2011). In this paper, we primarily investigated the possible role of VOC in the antixenosis phenomenon observed in the choice experiments. An antixenosis effect is also suggested by the EPG experiments on fundatrigeniae, which probed less and were less successful in phloem sap ingestion on ASM-treated plants. VOC emission is probably not responsible for the disturbed feeding behavior, because we did not observe any delay in the first stylet activity. Physico-chemical modifications of the tissues disturbing feeding behavior should therefore be considered. In this respect, gene expression data suggested that cell wall remodeling might occur after ASM treatment, with a possible consequence on stylet penetration.

The overexpression of terpene synthase-encoding gene promoted by ASM led to a substantial modification of the sesquiterpene blend emitted by apple plants, secondary metabolites which have already been shown to modify host preferences of insects, and their feeding behavior (Unsicker et al., 2009; Kamolsukyunyong et al., 2013; Bhatia et al., 2015). ASM treatment leads to a particular important emission of (*E,E*)-α-farnesene, for which we have also shown a dose-dependent repellent effect on *Dysaphis plantaginea* gynoparae. Emission of the same secondary metabolite has previously been shown to be induced upon herbivore attack in different plants, including apple and soybean (Boevé et al., 1996; Moraes et al., 2005; Lin et al., 2017), and to reduce attraction of the coffee berry borer when added to coffee berries (Vega et al., 2017). But so far, to our knowledge no direct repellent effect of (*E,E*)-α-farnesene on insects has been shown, even if the isomer (*E*)-β-farnesene, the alarm pheromone of aphids, is a well-known repellent of different aphid species (Bruce et al., 2005; Webster et al., 2010; Zhou et al., 2016). Germacrene-D, another repellent terpene (Bruce et al., 2005; Webster et al., 2010) is also produced upon ASM treatment but unfortunately we were not able to evaluate its contribution to the odor blend affecting *Dysaphis plantaginea* by antixenosis. In any case, to get closer to a natural situation, we will have to evaluate the effect of complex olfactory cues emitted by ASM-treated plants on *Dysaphis plantaginea* host seeking behavior, either by using plants confined in containers connected to the olfactometer or by using an odor blend collected from ASM-treated and untreated plants.

In addition to resistance by antixenosis, we found that ASM induces resistance by antibiosis: larval and adult longevity, female fecundity and duration of larval development of *Dysaphis plantaginea* fundatrigeniae were all affected on ASM-treated apples. The microarray analysis shed light on a gene family strongly up-regulated upon ASM treatment and which code for proteins containing a single agglutinin domain, reminiscent of the amaranthin lectin initially described in *Amaranthus caudatus* (Rinderle et al., 1990). This similarity raises the question of their involvement in the resistance by antibiosis we observed in apple against *Dysaphis plantaginea*.

Plant lectins are defined as proteins possessing at least one non-catalytic domain that binds reversibly to a specific mono- or oligosaccharide (Peumans and Van Damme, 1995). According to their functional domains, plant lectins are classified in four major groups: merolectins, hololectins, chimerolectins, and superlectins; and according to their carbohydrate-binding specificity they are classified in 12 families (Peumans and Van Damme, 1995; Van Damme et al., 2008; Vandenborre et al., 2011). Chimerolectins comprise proteins that contain at least one carbohydrate-binding domain fused to another domain that exerts its proper biological activity, which can be very diverse (Lannoo and Van Damme, 2014). Mero- and hololectins are proteins that contain only one and two or more identical carbohydrate-binding domains, respectively. Based on these definitions, MdAGGs are merolectins, MdDiAGG1 to 4 are hololectins and MdDiAGG5 to 7 are chimerolectins (aerolisin domain). Several mero- and hololectins have insecticidal activities, and the best-studied example concerns the merolectin of *Galanthus nivalis*, which (i) assembles as a homo-tetramer, (ii) binds specifically to terminal mannose residues of *N*-glycans and (iii) promotes resistance toward insects when ectopically expressed in various crops (Hester and Wright, 1996; Vandenborre et al., 2011). Interestingly the amaranthin hololectin from *A. caudatus* has also demonstrated aphicidal activity in various crops when ectopically expressed under the control of a phloem-specific promoter (Guo et al., 2004; Wu et al., 2006; Xin et al., 2011), or when included in artificial diets (Sauvion, 1995). The *Rhizoctonia solani* agglutinin is a Gal- and GalNAc-specific merolectin, which is able to bind the luminal side of the midgut epithelium of the pea aphid *Acyrthosiphon pisum* and whose toxicity relies on its ability to bind these specific sugars (Hamshou et al., 2013). Our results showed (i) enhanced mortality of *Dysaphis plantaginea* fundatrigeniae without acute toxicity on ASM-treated apple plants, (ii) accumulation of MdAGGs in various ASM-treated tissues and (iii) putative binding of MdAGGs with the disaccharide Gal-GalNAc. The delayed antibiosis effects observed after ASM treatment in apple could be due to ingestion of MdAGGs devoid of domains responsible for acute toxicity, but hampering nutrient absorption. However, future studies need to validate this hypothesis.

Our description of apple amaranthin-like lectins completes the recent description performed by Dang et al. (2017), although it did not allow us to identify tri-agglutinin encoding genes. Surprisingly, all mono-agglutinin encoding genes escaped the automatic annotation pipeline used for GDDH13 annotation. The high under-prediction of these genes by the automatic pipeline could be explained by their low RNAseq coverage in naïve conditions and their distribution in regions rich in transposable elements, probably leading to frequent sequence masking during the annotation process.

Previous studies demonstrated that ASM promotes the resistance of apple to fire blight (*E. amylovora*) and to apple scab (*V. inaequalis*). Here, we report on an additional protection efficiency of ASM in apple, namely against the rosy apple aphid *Dysaphis plantaginea*. It is thus very promising to see that a same compound induces resistance against very different pests and diseases. The next step will be to confirm the action of ASM against *Dysaphis plantaginea* in the field in order to evaluate its potential as a real alternative for the use of insecticides, such as neonicotinoids. Our results showing effects of induced resistance on both fundatrigeniae and gynoparae suggest that this protection method could even target two phases of the aphid’s reproductive cycle.

## Author Contributions

RW, MG, PR, SyA, CG, M-NB, and AD planned and designed the research. RW, PR, SoA, SyA, NB, FB, RC, CG, CH, and AD performed the experiments. RW, MG, PR, SyA, SéA, NB, CG, M-NB, and AD analyzed the data. RW, MG, SyA, M-NB, and AD drafted the manuscript which was reviewed and revised by all authors.

## Conflict of Interest Statement

The authors declare that the research was conducted in the absence of any commercial or financial relationships that could be construed as a potential conflict of interest.

## Footnotes

1 https://www.rosaceae.org/

2 https://iris.angers.inra.fr/gddh13/
