# Supplementary material for: Acibenzolar-S-Methyl Reprograms Apple Transcriptome Toward Resistance to Rosy Apple Aphid
Source: Front Plant Sci. 2018 Dec 12;9:1795. doi: 10.3389/fpls.2018.01795 (PMC6299034; doi:10.3389/fpls.2018.01795)
Supplement: Supplementary file 2 [file Data_Sheet_1.pdf]

Supplementary Material

Acibenzolar-S-methyl reprograms apple transcriptome towards resistance to rosy apple aphid

Romain Warneys[1], Matthieu Gaucher[1], Philippe Robert[1], Sophie Aligon[1], Sylvia Anton[2], Sébastien Aubourg[1], Nicolas Barthes[3], Ferréol Braud[1], Raphaël Cournol[1], Christophe Gadenne[2], Christelle Heintz[1], Marie-Noëlle Brisset[1], Alexandre Degrave[1*]

*** Correspondence:** Alexandre Degrave: alexandre.degrave@agrocampus-ouest.fr

1 Supplementary Data

Supplementary Material should be uploaded separately on submission. Please include any supplementary data, figures and/or tables. All supplementary files are deposited to FigShare for permanent storage and receive a DOI.

Supplementary material is not typeset so please ensure that all information is clearly presented, the appropriate caption is included in the file and not in the manuscript, and that the style conforms to the rest of the article.

2 Supplementary Figures and Tables

For more information on Supplementary Material and for details on the different file types accepted, please see here. Figures, tables, and images will be published under a Creative Commons CC-BY licence and permission must be obtained for use of copyrighted material from other sources (including re-published/adapted/modified/partial figures and images from the internet). It is the responsibility of the authors to acquire the licenses, to follow any citation instructions requested by third-party rights holders, and cover any supplementary charges.

2.1 Supplementary Figures

Supplementary Figure 1 Phylogenetic analysis of *MdAGG*s CDS

Supplementary Figure 2 Identity matrices of apple agglutinins (promoter, CDS and protein)

Supplementary Figure 3 MdAGG protein sequence alignement

Supplementary Table 1 Statistical values related to Table 1, Figures 2, 3 and 7.

Supplementary Table 2 Direct effect of ASM application on *Dysaphis plantaginea* mortality

Supplementary Table 3 Selected genes from microarray data set

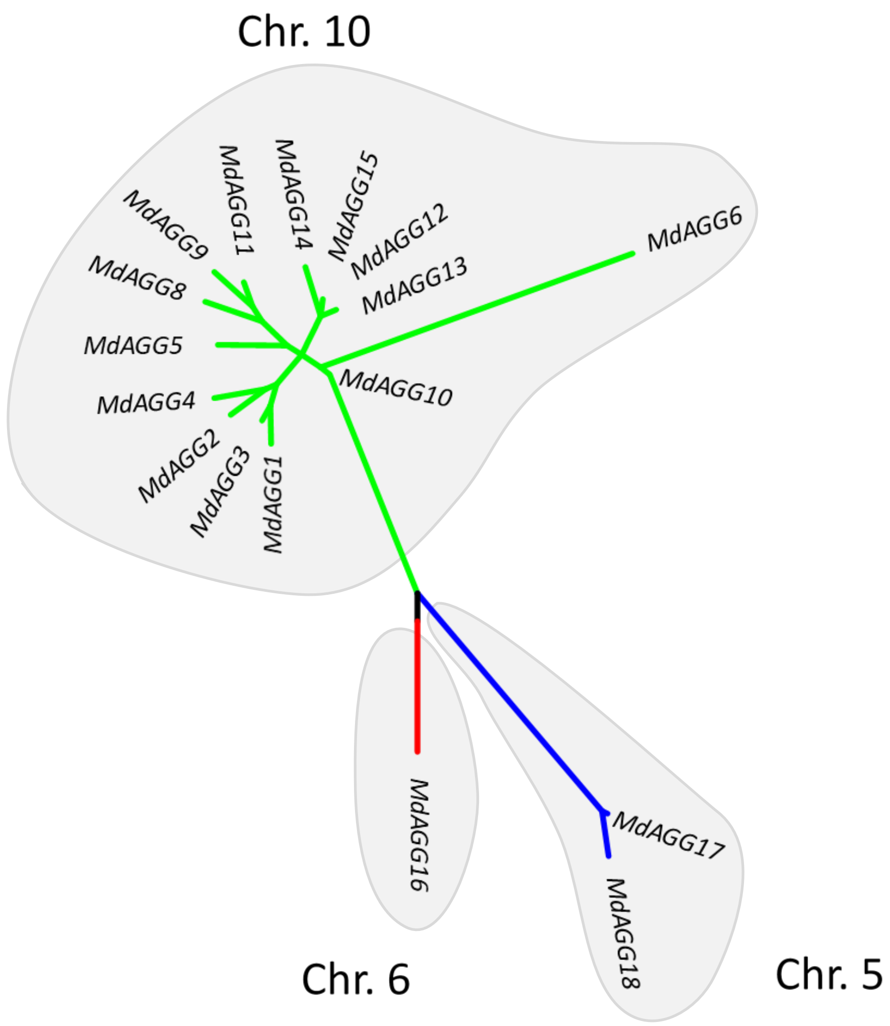

Figure S1 Phylogenetic analysis of *MdAGG*s CDS using the maximum likelihood method. The tree was built with MEGA7. Branches are colored according to the chromosome harboring the considered CDS.

(A)

Pairwise identity (%)

100
85
80
75
65
50

	MdDiAGG7	MdDiAGG5	MdDiAGG6	MdDiAGG3	MdDiAGG2	MdAGG17	MdAGG18	MdAGG16	MdAGG4	MdAGG2	MdAGG1	MdAGG3	MdAGG6	MdAGG5	MdAGG9	MdAGG11	MdAGG10	MdAGG8	MdAGG12	MdAGG13	MdAGG14	MdAGG15	MdDiAGG1	MdDiAGG4
MdDiAGG7		46.9	41.25	37.4	34.34	36.73	39.1	35.75	40.46	37.5	39.2	39.02	39.84	37.43	37.3	36.7	37.89	39.63	39.1	38.83	39.36	40.05	38.3	36.41
MdDiAGG5	46.9		54.63	37.08	35.34	33.68	32.09	33.51	32.13	33.87	34.77	33.24	34.65	34.04	34.93	34.05	35.62	36.19	36	36.27	35.47	36.27	36.76	37.4
MdDiAGG6	41.25	54.63		34.36	35.93	36.45	36.96	35.61	36.18	35.23	37.11	35.03	35.49	35.4	37.6	37.34	37.43	38.72	38.92	39.18	38.4	38.5	36.18	39
MdDiAGG3	37.4	37.08	34.36		43.97	43.13	42.2	45.23	42.07	42.55	43.93	42.53	46.02	42.55	43.83	43.1	41.91	43.1	43.34	43.34	42.62	43.1	39.28	41.27
MdDiAGG2	34.34	35.34	35.93	43.97		49.77	49.43	46.33	48.45	46.65	47.62	48.45	46.63	48.55	46.65	46.43	47.29	47.97	47.84	48.32	49.04	48.19	37.5	41.04
MdAGG17	36.73	33.68	36.45	43.13	49.77		94.8	58.72	62.53	59.5	61.22	64.07	57.67	59.4	58.31	59.64	60.37	60.68	61.1	60.87	61.33	61.01	40.14	41.75
MdAGG18	39.1	32.09	36.96	42.2	49.43	94.8		55.01	54.27	52.39	54.34	53.83	49.11	51.15	51.15	51.4	51.65	52.67	51.91	51.65	53.44	53.18	41.05	42.12
MdAGG16	35.75	33.51	35.61	45.23	46.33	58.72	55.01		68.36	73.92	76.07	74.83	68.85	71.74	73.99	74.04	75.93	75.69	75.97	76.18	74.89	75.91	37.38	40.74
MdAGG4	40.46	32.13	36.18	42.07	48.45	62.53	54.27	68.36		80.21	79.32	82.26	72.5	73.52	73.67	73.89	73.04	74.52	72.61	72.82	73.67	74.1	38.92	40.84
MdAGG2	37.5	33.87	35.23	42.55	46.65	59.5	52.39	73.92	80.21		91.02	91.83	76.41	79.46	81.15	81.56	80.92	81.97	79.67	79.88	81.52	81.48	40.62	41.65
MdAGG1	39.2	34.77	37.11	43.93	47.62	61.22	54.34	76.07	79.32	91.02		97.16	81.16	84.57	86.21	86.06	86.69	87.25	85.95	86.15	86.15	86.73	40.24	41.48
MdAGG3	39.02	33.24	35.03	42.53	48.45	64.07	53.83	74.83	82.26	91.83	97.16		79.34	82.94	85.02	85.68	84.36	85.9	84.36	84.36	85.24	85.24	40.3	42.34
MdAGG6	39.84	34.65	35.49	46.02	46.63	57.67	49.11	68.85	72.5	76.41	81.16	79.34		82.21	80.45	80.21	80	80.41	79.79	80	80.82	80.41	39.12	41.58
MdAGG5	37.43	34.04	35.4	42.55	48.55	59.4	51.15	71.74	73.52	79.46	84.57	82.94	82.21		87.32	87.09	85.77	86.89	85.66	85.66	86.89	86.48	40.67	42.01
MdAGG9	37.3	34.93	37.6	43.83	46.65	58.31	51.15	73.99	73.67	81.15	86.21	85.02	80.45	87.32		96.79	94.06	93.57	92.34	92.54	93.35	93.33	38.86	40.97
MdAGG11	36.7	34.05	37.34	43.1	46.43	59.64	51.4	74.04	73.89	81.56	86.06	85.68	80.21	87.09	96.79		92.59	91.58	90.93	91.13	91.53	91.11	38.1	40.71
MdAGG10	37.89	35.62	37.43	41.91	47.29	60.37	51.65	75.93	73.04	80.92	86.69	84.36	80	85.77	94.06	92.59		96.3	94.89	95.3	96.32	96.32	38.98	39.58
MdAGG8	39.63	36.19	38.72	43.1	47.97	60.68	52.67	75.69	74.52	81.97	87.25	85.9	80.41	86.89	93.57	91.58	96.3		94.97	95.17	95.98	96.37	39.05	40.31
MdAGG12	39.1	36	38.92	43.34	47.84	61.1	51.91	75.97	72.61	79.67	85.95	84.36	79.79	85.66	92.34	90.93	94.89	94.97		99.4	95.6	95.59	37.95	41.03
MdAGG13	38.83	36.27	39.18	43.34	48.32	60.87	51.65	76.18	72.82	79.88	86.15	84.36	80	85.66	92.54	91.13	95.3	95.17	99.4		95.8	95.79	37.95	41.03
MdAGG14	39.36	35.47	38.4	42.62	49.04	61.33	53.44	74.89	73.67	81.52	86.15	85.24	80.82	86.89	93.35	91.53	96.32	95.98	95.6	95.8		97.6	38.9	40.77
MdAGG15	40.05	36.27	38.5	43.1	48.19	61.01	53.18	75.91	74.1	81.48	86.73	85.24	80.41	86.48	93.33	91.11	96.32	96.37	95.59	95.79	97.6		39.23	40.77
MdDiAGG1	38.3	36.76	36.18	39.28	37.5	40.14	41.05	37.38	38.92	40.62	40.24	40.3	39.12	40.67	38.86	38.1	38.98	39.05	37.95	37.95	38.9	39.23		54.74
MdDiAGG4	36.41	37.4	39	41.27	41.04	41.75	42.12	40.74	40.84	41.65	41.48	42.34	41.58	42.01	40.97	40.71	39.58	40.31	41.03	41.03	40.77	40.77	54.74	

(B)

Pairwise identity (%)

100
95
90
85
75
50

	MdDiAGG2	MdDiAGG1	MdDiAGG4	MdDiAGG3	MdAGG17	MdAGG18	MdAGG16	MdAGG6	MdAGG8	MdAGG9	MdAGG11	MdAGG4	MdAGG2	MdAGG1	MdAGG3	MdAGG5	MdAGG10	MdAGG14	MdAGG12	MdAGG13	MdAGG15	MdDiAGG7	MdDiAGG5	MdDiAGG6
MdDiAGG2		79.65	76.01	59.15	68.67	68.47	67.27	64.46	66.67	67.67	67.47	67.87	67.67	67.67	68.07	67.67	67.87	67.07	66.47	67.47	67.47	45.13	44.76	44.69
MdDiAGG1	79.65		81.73	59.25	70.48	70.08	69.88	66.87	68.48	69.88	70.08	70.28	69.88	69.88	70.28	69.68	70.68	69.88	68.66	69.88	69.88	47.76	46.26	46.3
MdDiAGG4	76.01	81.73		60.96	68.27	68.27	67.47	66.06	66.26	67.27	67.67	67.87	68.07	67.87	68.27	67.67	68.47	67.67	67.07	67.67	67.67	47.32	47.87	46.88
MdDiAGG3	59.15	59.25	60.96		73.33	73.13	73.01	69.49	71.81	71.37	71.78	71.78	71.78	71.78	71.57	71.57	72.19	71.57	71.54	71.37	71.78	41.64	39.81	40.31
MdAGG17	68.67	70.48	68.27	73.33		98.81	90.16	84.33	86.46	86.95	87.55	88.15	88.35	89.16	88.96	88.55	88.96	87.55	87.35	87.95	88.35	38.16	36.04	36.71
MdAGG18	68.47	70.08	68.27	73.13	98.81		89.36	83.53	85.66	86.14	86.75	87.55	87.95	88.76	88.55	88.15	88.15	87.15	86.95	87.55	87.95	38.38	36.04	36.26
MdAGG16	67.27	69.88	67.47	73.01	90.16	89.36		86.14	87.27	88.76	88.96	89.56	89.76	90.16	90.36	89.96	91.16	89.76	89.16	89.76	90.16	38.44	36.07	36.76
MdAGG6	64.46	66.87	66.06	69.49	84.33	83.53	86.14		88.69	90.36	90.56	91.16	90.96	90.76	91.37	91.16	92.57	90.76	90.76	91.77	91.37	40.12	37.76	39.03
MdAGG8	66.67	68.48	66.26	71.81	86.46	85.66	87.27	88.69		94.95	95.96	93.33	93.94	93.74	93.94	94.55	94.95	93.33	94.34	93.94	94.34	38.03	35.86	37.47
MdAGG9	67.67	69.88	67.27	71.37	86.95	86.14	88.76	90.36	94.95		98.19	95.58	95.38	94.98	95.58	96.79	96.39	95.38	95.18	95.78	96.18	39.33	37.21	37.9
MdAGG11	67.47	70.08	67.67	71.78	87.55	86.75	88.96	90.56	95.96	98.19		95.78	96.18	95.58	96.18	96.59	96.99	95.58	95.78	96.39	96.79	39.56	37.21	38.81
MdAGG4	67.87	70.28	67.87	71.78	88.15	87.55	89.56	91.16	93.33	95.58	95.78		97.79	97.39	97.59	96.79	96.79	95.38	95.58	96.18	96.18	38.67	36.53	38.81
MdAGG2	67.67	69.88	68.07	71.78	88.35	87.95	89.76	90.96	93.94	95.38	96.18	97.79		97.99	97.79	96.59	96.99	95.58	95.78	96.39	96.39	38.22	36.3	38.36
MdAGG1	67.67	69.88	67.87	71.78	89.16	88.76	90.16	90.76	93.74	94.98	95.58	97.39	97.99		98.8	96.99	96.99	95.98	95.78	96.39	96.59	38	36.76	38.58
MdAGG3	68.07	70.28	68.27	71.57	88.96	88.55	90.36	91.37	93.94	95.58	96.18	97.59	97.79	98.8		96.79	97.59	96.59	96.39	96.99	97.19	38.67	36.76	39.27
MdAGG5	67.67	69.68	67.67	71.57	88.55	88.15	89.96	91.16	94.55	96.79	96.59	96.79	96.59	96.99	96.79		97.19	96.18	95.98	96.59	96.99	38.44	37.21	37.21
MdAGG10	67.87	70.68	68.47	72.19	88.96	88.15	91.16	92.57	94.95	96.39	96.99	96.79	96.99	96.99	97.59	97.19		96.99	97.19	97.79	97.79	38.89	36.53	38.58
MdAGG14	67.07	69.88	67.67	71.57	87.55	87.15	89.76	90.76	93.33	95.38	95.58	95.38	95.58	95.98	96.59	96.18	96.99		97.79	98.39	98.39	39.33	37.67	38.81
MdAGG12	66.47	68.66	67.07	71.54	87.35	86.95	89.16	90.76	94.34	95.18	95.78	95.58	95.78	95.78	96.39	95.98	97.19	97.79		98.59	98.59	39.07	37.19	38.55
MdAGG13	67.47	69.88	67.67	71.37	87.95	87.55	89.76	91.77	93.94	95.78	96.39	96.18	96.39	96.39	96.99	96.59	97.79	98.39	98.59		99.2	38.89	37.44	39.04
MdAGG15	67.47	69.88	67.67	71.78	88.35	87.95	90.16	91.37	94.34	96.18	96.79	96.18	96.39	96.59	97.19	96.99	97.79	98.39	98.59	99.2		39.33	37.67	38.81
MdDiAGG7	45.13	47.76	47.32	41.64	38.16	38.38	38.44	40.12	38.03	39.33	39.56	38.67	38.22	38	38.67	38.44	38.89	39.33	39.07	38.89	39.33		57.77	57.89
MdDiAGG5	44.76	46.26	47.87	39.81	36.04	36.04	36.07	37.76	35.86	37.21	37.21	36.53	36.3	36.76	36.76	37.21	36.53	37.67	37.19	37.44	37.67	57.77		86.17
MdDiAGG6	44.69	46.3	46.88	40.31	36.71	36.26	36.76	39.03	37.47	37.9	38.81	38.81	38.36	38.58	39.27	37.21	38.58	38.81	38.55	39.04	38.81	57.89	86.17	

(C)

Pairwise identity (%)

100
95
90
85
75
50

	MdDiAGG2	MdDiAGG1	MdDiAGG4	MdDiAGG3	MdAGG6	MdAGG17	MdAGG18	MdAGG16	MdAGG8	MdAGG12	MdAGG14	MdAGG13	MdAGG15	MdAGG9	MdAGG11	MdAGG4	MdAGG5	MdAGG10	MdAGG2	MdAGG1	MdAGG3	MdDiAGG7	MdDiAGG5	MdDiAGG6
MdDiAGG2		65.31	60	41.36	44.24	50.91	51.52	50.3	48.17	48.19	49.09	49.09	48.48	49.09	49.09	47.88	48.48	48.48	48.48	49.09	49.09	30.2	32.2	31.29
MdDiAGG1	65.31		69.57	42.37	47.27	53.33	52.73	52.12	51.83	51.81	52.12	52.73	52.12	53.33	53.33	51.52	52.12	52.12	51.52	51.52	51.52	32.65	32.99	33.1
MdDiAGG4	60	69.57		42.03	44.85	51.52	50.91	49.09	48.17	48.8	49.09	48.48	49.09	49.7	49.7	48.48	49.09	49.09	49.09	49.09	49.09	32.31	34.36	34.14
MdDiAGG3	41.36	42.37	42.03		57.32	60.98	60.37	62.96	62.11	61.35	61.73	61.11	61.73	61.73	61.73	62.35	61.73	61.11	61.73	62.35	61.73	22.63	23.25	22.59
MdAGG6	44.24	47.27	44.85	57.32		79.04	77.84	84.24	82.93	84.24	85.45	86.06	85.45	84.85	84.85	86.06	86.67	86.67	86.67	87.27	86.67	15.38	21.33	20.67
MdAGG17	50.91	53.33	51.52	60.98	79.04		98.8	89.7	85.37	86.67	88.48	87.27	87.88	87.27	87.27	88.48	89.09	88.48	89.09	90.3	90.91	16.44	21.28	21.99
MdAGG18	51.52	52.73	50.91	60.37	77.84	98.8		88.48	84.15	85.45	87.27	86.06	86.67	86.06	86.06	87.88	87.88	87.27	87.88	89.09	89.7	16.44	21.28	21.99
MdAGG16	50.3	52.12	49.09	62.96	84.24	89.7	88.48		89.02	91.52	93.33	92.12	92.73	90.91	90.91	92.12	92.73	92.73	92.73	93.94	93.33	16.67	23.02	22.3
MdAGG8	48.17	51.83	48.17	62.11	82.93	85.37	84.15	89.02		94.51	94.51	93.9	94.51	96.34	96.34	93.9	95.73	95.12	94.51	95.12	94.51	16.08	21.01	21.74
MdAGG12	48.19	51.81	48.8	61.35	84.24	86.67	85.45	91.52	94.51		98.18	98.18	98.79	95.76	95.76	95.76	97.58	96.97	96.36	96.36	95.76	17.24	22.14	22.86
MdAGG14	49.09	52.12	49.09	61.73	85.45	88.48	87.27	93.33	94.51	98.18		98.79	99.39	96.36	96.36	96.36	98.18	97.58	96.97	98.18	97.58	17.36	22.3	23.02
MdAGG13	49.09	52.73	48.48	61.11	86.06	87.27	86.06	92.12	93.9	98.18	98.79		99.39	96.36	96.36	96.36	98.18	97.58	96.97	96.97	96.36	17.36	22.3	23.02
MdAGG15	48.48	52.12	49.09	61.73	85.45	87.88	86.67	92.73	94.51	98.79	99.39	99.39		96.97	96.97	96.97	98.79	98.18	97.58	97.58	96.97	17.36	22.3	23.02
MdAGG9	49.09	53.33	49.7	61.73	84.85	87.27	86.06	90.91	96.34	95.76	96.36	96.36	96.97		100	96.36	98.18	97.58	96.97	96.97	96.36	17.36	22.3	23.02
MdAGG11	49.09	53.33	49.7	61.73	84.85	87.27	86.06	90.91	96.34	95.76	96.36	96.36	96.97	100		96.36	98.18	97.58	96.97	96.97	96.36	17.36	22.3	23.02
MdAGG4	47.88	51.52	48.48	62.35	86.06	88.48	87.88	92.12	93.9	95.76	96.36	96.36	96.97	96.36	96.36		98.18	97.58	98.18	98.18	97.58	18.06	23.02	24.46
MdAGG5	48.48	52.12	49.09	61.73	86.67	89.09	87.88	92.73	95.73	97.58	98.18	98.18	98.79	98.18	98.18	98.18		99.39	98.79	98.79	98.18	17.36	22.3	23.02
MdAGG10	48.48	52.12	49.09	61.11	86.67	88.48	87.27	92.73	95.12	96.97	97.58	97.58	98.18	97.58	97.58	97.58	99.39		98.18	98.18	97.58	17.36	22.3	22.3
MdAGG2	48.48	51.52	49.09	61.73	86.67	89.09	87.88	92.73	94.51	96.36	96.97	96.97	97.58	96.97	96.97	98.18	98.79	98.18		98.79	98.18	17.36	21.58	22.3
MdAGG1	49.09	51.52	49.09	62.35	87.27	90.3	89.09	93.94	95.12	96.36	98.18	96.97	97.58	96.97	96.97	98.18	98.79	98.18	98.79		99.39	17.36	22.3	23.02
MdAGG3	49.09	51.52	49.09	61.73	86.67	90.91	89.7	93.33	94.51	95.76	97.58	96.36	96.97	96.36	96.36	97.58	98.18	97.58	98.18	99.39		17.36	22.3	23.02
MdDiAGG7	30.2	32.65	32.31	22.63	15.38	16.44	16.44	16.67	16.08	17.24	17.36	17.36	17.36	17.36	17.36	18.06	17.36	17.36	17.36	17.36	17.36		43.6	41.61
MdDiAGG5	32.2	32.99	34.36	23.25	21.33	21.28	21.28	23.02	21.01	22.14	22.3	22.3	22.3	22.3	22.3	23.02	22.3	22.3	21.58	22.3	22.3	43.6		78.92
MdDiAGG6	31.29	33.1	34.14	22.59	20.67	21.99	21.99	22.3	21.74	22.86	23.02	23.02	23.02	23.02	23.02	24.46	23.02	22.3	22.3	23.02	23.02	41.61	78.92	

Figure S2 Color coded pairwise identity (%) matrix of MdAGGs and MdDiAGGs. These matrices are based on (A) promoter sequences encompassing the 500 bp upstream of start codon, (B) CDS and (C) protein sequences. Each color cell represents a percent identity score between two sequences. A colored key indicates the correspondence between pairwise identities and colors displayed in the matrix.

```
                                                                  50
β-sheet (1jlx)
AcaAGG (1jlx)     MAGLPVI---MCLKSNNNQKYLRYQSDNIQQYG-LLQFSADKILDPLAQF
MdAGG1            -MAPPVLPSPFLLKADTNNKYLRYQLDAESDLNEIVQFSEDNENSRFIKF
MdAGG2            -MAPPVLPSPFLLKADTNNKYLRYQLDAESDLNEIVQFSEDNENSRFIKF
MdAGG3            -MAPPVLPSPFLLKADTNNKYLRYQLDAESDLNEIVQFSEDNENSRFVKF
MdAGG4            -MAPPVLPSPFLLKADTNNKYLRYQLDAESDLNEIVQFSEDNENSRFIKF
MdAGG5            -MAPPVLPSPFLLKADINNKYLRYQLDAESDLNEIVQFSEDNENSRFIKF
MdAGG6            -MAPPVLPSPFLLKANTKNKYLRYQLDAESDLNEIVQFFEDNENSRFIKF
MdAGG8            -MAPPV-PSPFLLKADINNKYLRYQLDSESDLHEIVQFSEDNENSRFIKF
MdAGG9            -MAPPVLPSPFLLKADINNKYLRYQLDSESDLHEIVQFSEDNENSRFIKF
MdAGG10           -MAPPVLPSPFLLKADINNKYLRYQLDAESDLNEIVQFSEDNENSRFIKF
MdAGG11           -MAPPVLPSPFLLKADINNKYLRYQLDSESDLHEIVQFSEDNENSRFIKF
MdAGG12           MAPPPVLPSPFLLKADINNKYLRYQLDAESDLNEIVQFSEDNENSRFIKF
MdAGG13           -MAPPVLPSPFLLKADINNKYLRYQLDAESDLNEIVQFSEDNENSRFIKF
MdAGG14           -MAPPVLPSPFLLKADINNKYLRYQLDAESDLNEIVQFSEDNENSRFIKF
MdAGG15           -MAPPVLPSPFLLKADINNKYLRYQLDAESDLNEIVQFSEDNENSRFIKF
MdAGG16           -MAPPVLPSPFLLKAETNNKYLRYQLDAESDLNEIVQFSEDNPNSRFIKF
MdAGG17           -MAPPVLPSPFLLKAETNNKYLRYQLDAESDINEIVQFSEDNPDSRFVKF
MdAGG18           -MAPPVLPSPFLLKAETNNKYLRYQLDAESDINEIVQFSEDNPDSRFVKF
synthetic peptide                   NKYLRYQLDAESDLN
MdDiAGG1                            NKYLRYMLDEENQTH 9/15
MdDiAGG2                            DKYLSYILDEESQNH 8/15
MdDiAGG3                            NKYLRYQSDVESGTD 10/15
MdDiAGG4                            NRYLRYMLDQENNIH 8/15
MdDiAGG5                            HYLGARLIDQYPSLL 2/15
MdDiAGG6                            NYLSARLIDQQPHLL 3/15
MdDiAGG7                            SYVTAHMADRNHMDH 1/15

                                                                 100
β-sheet (1jlx)
AcaAGG (1jlx)     EVEPSKTYD----GLVHIKSRYTNKYLVRWSPNHYWITASANEPDENKSN
MdAGG1            TTEKPNNEDYADKNYVHIKCSYNGNYLRRVDQNRLLVLAAAADRNETKDN
MdAGG2            TTEKPNNEDYADKNYVHIKCSYNSNYLRRVDQNRLLVLAAAADRNETKDN
MdAGG3            TTEKPNNEDYADKNYVHIKCSYNGNYLRRVDQNRLLVLAAAADRNETKDN
MdAGG4            TTEKPNNEDYADKNYVHIKCSYNGNYLRRVDQNRLLVLAAAADRNETKDN
MdAGG5            TTEKPNNEDYADKNYVHIKCSYNGNYLRRVDQNRLLVLAAAADRNETKDN
MdAGG6            ITEKPNNEDYADKNYVHIKCSYIGNYLRRVDQNKLLVLAAAADQNETKDN
MdAGG8            TTEKPNNEDYADKNYVHIKCSYNGNYLRRVDQNRLLVLAAAADRNETKDN
MdAGG9            TTEKPNNEDYADKNYVHIKCSYNGNYLRRVDQNRLLVLAAAADRNETKDN
MdAGG10           TTEKPNNEDYADKNYVHIKCSYNGNYLRRVDQNRLLVLAAAADRNETKDN
MdAGG11           TTEKPNNEDYADKNYVHIKCSYNGNYLRRVDQNRLLVLAAAADRNETKDN
MdAGG12           TTEKPNNEDYADKNYVHIKCSYNGNYLRRVDQNRLLVLAAAADRNETKDN
MdAGG13           TTEKPNNEDYADKNYVHIKCSYNGNYLRRVDQNKLLVLAAAADRNETKDN
MdAGG14           TTEKPNNEDYADKNYVHIKCSYNGNYLRRVDQNRLLVLAAAADRNETKDN
MdAGG15           TTEKPNNEDYADKNYVHIKCSYNGNYLRRVDQNRLLVLAAAADRNETKDN
MdAGG16           TTEKPNNEDYADKNYVHIKCSYNGNYLRRVDQNRLLVLAAAADRNETKDN
MdAGG17           TTETPNNEDYADKHYVHIKCSYNGNYLRRVDQNRLLVLAAATDRNETKDN
MdAGG18           TTETPNNEDYADKHYVHIKCSYNGNYLRRVDQNRLLVLAVATDRNETKDN
synthetic peptide  TEKPNNEDYADKNYV
MdDiAGG1           VEQANNQDDQDNHYV 7/15
MdDiAGG2           VEQANSKDHKENHYV 5/15
MdDiAGG3           TEKPNNKDYADQNYV 13/15
MdDiAGG4           VERANSRDHQNNHYV 5/15
MdDiAGG5           NADGTVRIKSDSSGK 1/15
MdDiAGG6           NADGTVRIKSNSSGK 0/15
MdDiAGG7           NPDGTLQIKNSFFER 0/15

                                                                 150
synthetic peptide
β-sheet (1jlx)
AcaAGG (1jlx)     WACTLFK--PLYVEEGN--MKKVRLLHVQLGHYTQNYTVGGSFVSYLFAE
MdAGG1            WACTLFKVEPVGPPDSNNLITRCRLRHLQSDLLTRPF-IENRFELRLNQK
MdAGG2            WACTLFKVEPVGPPDSNNLITRCRLRHLQSDLLTRPF-IENRFELRLNQK
MdAGG3            WACTLFKVEPVGPPDSNNLITRCRLRHLQSDLLTRPF-IENRFELRLNQK
MdAGG4            WACTLFKVEPVGPPDSNNLITRCRLRHLQSNLLTRPF-IESRFELRLNQK
MdAGG5            WACTLFKVEPVGPPDSNNLITRCRLRHLQSDLLTRPF-IENRFELRLNQK
MdAGG6            WACTLFKVEPVEPPNSNNLITRCRLRHLQTDLLTTPF-IENIFELSLNQK
MdAGG8            WACTLFKVEPVGPPDSNNLITRCRLRHLQSDLLTRPF-IENRFELRLNQK
MdAGG9            WACTLFKVEHVGPPDSNNLITRCRLRHLQSDLLTRPF-IENRFELRLNQK
MdAGG10           WACTLFKVEPVGPPDSNNLITRCRLRHLQSDLLTRPF-IENRFELRLNQK
MdAGG11           WACTLFKVEHVGPPDSNNLITRCRLRHLQSDLLTRPF-IENRFELRLNQK
MdAGG12           WACTLFKVEPVGPPDSNNLITRCRLRHLQSDLITRPF-IENRFELRLNQK
MdAGG13           WACTLFKVEPVGPPDSNNLITRCRLRHLQSDLITRPF-IENRFELRLNQK
MdAGG14           WACTLFKVEPVGPPDSNNLITRCRLRHLQSDLITRPF-IENRFELRLNQK
MdAGG15           WACTLFKVEPVGPPDSNNLITRCRLRHLQSDLITRPF-IENRFELRLNQK
MdAGG16           WACTLFKVEPVGPPDGNNLITRCRLRHLQSDLVTRPF-IENRFELRLNKK
MdAGG17           WACTLFKVEPVGPPDNNNQITRCRLRHLQSDLLTRPF-IENRFELRLHQK
MdAGG18           WACTLFKVEPVGPPDNNNQITRCRLRHLQSYLLTRPF-IENRFELRLHQK

                                                                 200
β-sheet (1jlx)
AcaAGG (1jlx)     SSQIDTGSKDVFHVIDWKSIFQFPKRYVTFKGNNGKYLGVITINQLPCLQ
MdAGG1            TP--DAGGVDIY-----------------------------SVSQGRC--
MdAGG2            TP--DAGGVDIY-----------------------------SVSQVRC--
MdAGG3            TP--DAGGVDIY-----------------------------SVSQGRC--
MdAGG4            TP--DAGGVDIY-----------------------------SVSQVRC--
MdAGG5            TP--DAGGVDIY-----------------------------SVSQVRC--
MdAGG6            TH--DARGVDIY-----------------------------QLLIHKCIS
MdAGG8            TP--DAGGVDIY-----------------------------SVSQARC--
MdAGG9            TP--DAGGVDIY-----------------------------SVSQVRC--
MdAGG10           TP--DAGGVDIY-----------------------------SVFQVRC--
MdAGG11           TP--DAGGVDIY-----------------------------SVSQVRC--
MdAGG12           QP--DAGGVDIY-----------------------------SVSQVRC--
MdAGG13           QP--DAGGVDIY-----------------------------SVSQVRC--
MdAGG14           QP--DAGGVDIY-----------------------------SVSQGRC--
MdAGG15           QP--DAGGVDIY-----------------------------SVSQVRC--
MdAGG16           IP--DSGGVDIY-----------------------------SVTCGKC--
MdAGG17           TP--DSQGVDMY-----------------------------SVSGGTCKC
MdAGG18           TP--DSQGVDMY-----------------------------SVSGGTCKC

AcaAGG            FGYDNLNDPKVAH (sequence to be continued for AcaAGG -1jlx)
MdAGG1            -------------
MdAGG2            -------------
MdAGG3            -------------
MdAGG4            -------------
MdAGG5            -------------
MdAGG6            NRAFKSKPKK---
MdAGG8            -------------
MdAGG9            -------------
MdAGG10           -------------
MdAGG11           -------------
MdAGG12           -------------
MdAGG13           -------------
MdAGG14           -------------
MdAGG15           -------------
MdAGG16           -------------
MdAGG17           -------------
MdAGG18           -------------
```

Figure S3 (previous page) Muscle protein sequence alignment of apple and *A. caudatus* (AcaAGG) mono-agglutinins. Conserved residues are in yellow. Beta-sheets of 1jlx are indicated in blue. Residues involved in the interaction between AcaAGG and ligand are in red, bold and underlined; those involved in the predicted interaction between MdAGGs and Gal-GalNAc are in bold. Residues from the synthetic peptide are indicated in green when identical and in red when different. Portions of MdDiAGGs are aligned to this sequence in order to visualize sequence identities (indicated on the right-hand side) using the same color code.

Table S1 Statistical values for the analysis of plant choice tests (related to Table 1), fundatrigeniae life traits (related to figure 2), EPG analysis (related to Figure 3) and olfactometer experiments on gynoparae (related to Figure 7).

**Plant Choice Tests (Mann and Withney)**		
**Morph**	**W-value**	**P-value**
**Fundatrigenia**	25	0.01167
**Gynoparae**	16	0.02652
**Fecundity per surviving adult fundatrigenia (Generalized linear mixed models, Poisson)**		
**Factor**	**χ^2**	**p-value**
**Treatment**	103.81	< 2.2e-16
**Time**	430.217	< 2.2e-16
**Intersection**	44.861	1.46e-7
**Survival analysis (Cox regression survival analysis)**		
**Experiment**	**Z-value**	**p-value**
**Adult longevity**	-2.296	0.0217
**Larval development (adults)**	5.541	3.01e-8
**Larval development (mortality)**	-10.8	< 2.2e-16
**Olfactometer experiment (paired t-test)**		
**Farnesene dose**	**T-value**	**p-value**
**10 ng**	0.993	0.328
**100 ng**	-1.64	0.111
**1 µg**	-4.146	0.00026
**10 µg**	-3.544	0.001
**100 µg**	-0.881	0.385
**Olfactometer experiment (paired t-test)**		
**Caryophyllene dose**	**T-value**	**p-value**
**10 ng**	-1.199	0.239
**100 ng**	0.069	0.946
**1 µg**	-1.159	0.255
**10 µg**	-0.991	0.322
**Olfactometer experiment (paired t-test)**		
**Blend Farnese Caryophyllene**	**T-value**	**p-value**
**03:01**	-0.905	0.372
**Control**	**T-value**	**p-value**
**Two-control**	-0.727	0.473
**EPG analysis (Mann and Withney)**		
**Item**	**W-value**	**p-value**
**#T**	443	0.0102
**#np**	265	0.3799
**#C**	380.5	0.1756
**#E1**	479.5	0.0010
**#E2**	549	0.0000

**Td np**	183	0.0125
**Td C**	277	0.5240
**Td E1**	270	0.4361
**Td E2**	494.5	0.0003
**Ad np**	192	0.0207
**Ad C**	216	0.0670
**Ad E1**	155	0.0025
**Ad E2**	453.5	0.0048
**T> 1st E1**	201	0.0339
**T > 1st E2**	83	0.0000
**D 1st E1**	317	0.9070
**D 1st E2**	457.5	0.0037
**T > 1st C**	348	0.4748
**D 1st C**	319	0.8774
**1 st E1<T> 1st E2**	108.5	0.0001
**# np > 1st E1**	233	0.1326
**Td np > 1st E1**	204	0.0383
**# C > 1st E1**	237	0.1538
**Td C > 1st E1**	172	0.0065

Table S2 Direct effect of ASM application on *Dysaphis plantaginea* fundatrigeniae mortality. Method as described in Robert *et al*., 2016 with ASM (a.i. of Bion® 50 WG) at 0.2 g l^{-1}.

Observations (3 days after spraying)	control	ASM
Total no.aphids	100	100
% living aphids	84	91
% dead aphids	12	4
% missing aphids	4	5
χ^2/d.f./p-value	4.39/2/0.11	
